# Pregnancy and the Eye

**DOI:** 10.4274/tjo.43815

**Published:** 2015-10-05

**Authors:** Nursal Melda Yenerel, Raciha Beril Küçümen

**Affiliations:** 1 Haydarpaşa Research and Training Hospital, Clinic of Eye, İstanbul, Turkey; 2 Yeditepe University Faculty of Medicine, Department of Ophthalmology, İstanbul, Turkey

**Keywords:** Ocular diseases in pregnancy, treatment of the eye during pregnancy, Eye, Pregnancy

## Abstract

Pregnancy causes significant changes in all systems of the body. Although most of them are physiological, they may also lead to pathological consequences. The resulting pathological changes may occur for the first time or existing diseases affected by pregnancy can become more serious or change course. Diseases specific only to pregnancy may arise. Like all systems of the body, the visual system is also affected by pregnancy, developing a wide range of physiological and pathological changes. Knowing the ocular physiological changes and diagnosing eye diseases that may develop during pregnancy, and preventing and treating these diseases is crucial to ensure the baby’s healthy development. Therefore, we have reviewed the conditions that an ophthalmologist should recognize, follow-up, and pay attention to during treatment and summarized them under the topic “pregnancy and the eye”.

## INTRODUCTION

Pregnancy causes major changes in all systems of the body. Physiological changes protect the fetus, support development, and also prepare the mother for birth. These changes affect the cardiovascular, renal, pulmonary, endocrine, metabolic, hematologic and visual systems.^1^ For example, in early pregnancy cardiac output and blood volume increase by 30-50%. Decreased fibrinolytic activity and increases in plasminogen, fibrinogen and factors I, V, VII, IX and X result in a predisposition toward coagulation. Toward the end of pregnancy, extracellular fluid increases by up to two liters. Cellular immunity decreases, but there are no changes in immunoglobins.^2^

Ocular changes during pregnancy are categorized as physiological or pathological. Pregnancy-related pathological changes may present as new ocular developments, changes in existing ocular pathology, and ocular complications of systemic diseases.^3,4^

This article examines pregnancy-related physiological and pathological changes in the eye and visual system; the diagnosis, monitoring and treatment of these changes; and issues to be considered.

## PHYSIOLOGICAL OCULAR CHANGES

The most frequent pregnancy-related physiological change is an increase in pigmentation around the eyes. Darkening of the face during pregnancy is referred to as pregnancy mask, cloasma or melasma and develops through increased estrogen, progesterone and melanocyte-stimulating hormone.^5^ Unilateral ptosis has been reported during pregnancy and following normal delivery. Ptosis is believed to develop as a result of fluid and hormonal effects on the levator aponeurosis, and it resolves postpartum.^6^

A decrease in conjunctival capillaries and an increase in granularity in conjunctival venules may occur, and they also resolve in the postpartum period.^3^

Pregnancy can also affect tear film physiology and lead to dry eye. This may be attributable to increased immune reaction in the lacrimal duct cells and the direct destruction of acinar cells by prolactin, transforming growth factor beta-1 and epidermal growth factor. Dryness can be further increased by dehydration resulting from nausea and vomiting and the use of anti-nausea medications.^7^

During pregnancy there may be a decrease in the sensitivity of the cornea which becomes more evident toward the end of pregnancy.^8^ The cornea thickens in response to corneal edema. Alterations in corneal curvature may occur, increasing in late pregnancy and resolving after the conclusion of the birth and breastfeeding period.^9^ Edema-related changes in corneal thickness and refractive index may occur, which therefore affects refraction.^10^ Changes in the cornea and lacrimal system during pregnancy may lead to contact lens intolerance.^11^

Increase in lens curvature may cause myopic shift. Temporary accommodation loss and insufficiency during pregnancy and the postnatal breastfeeding period have been reported. Therefore, new eyeglass and contact lens prescriptions should be avoided during pregnancy, and are best postponed until several months after delivery. Refractive surgery is contraindicated during pregnancy.^3^

Krukenberg’s spindle may appear in the first two trimesters; with increasing ease of outflow in the last trimester and postpartum, the spindle shrinks and disappears.

Intraocular pressure (IOP) decreases during pregnancy. A 19.6% reduction for individuals with normal IOP and a 24.4% reduction for ocular hypertension patients have been reported. Various mechanisms have been proposed to explain pregnancy-related IOP reduction, including increased aqueous outflow, lower episcleral venous pressure due to decreased systemic vascular resistance, lower scleral rigidity as a result of increased tissue elasticity, and general acidosis during pregnancy.^12,13,14^ Existing glaucoma typically improves during pregnancy, although there are reports of cases in which IOP was difficult to regulate.^15,16,17^ Pregnant patients may not want to use glaucoma medication because of their teratogenic effects; in that situation, the need for medication can be lessened with laser trabeculoplasty before the patient plans to become pregnant.^16,17^ During normal pregnancy, no physiological changes in the retinal arterioles, venules and capillary bed have been observed.

Changes in the visual field may occur. The pituitary gland grows physiologically during pregnancy; this can lead to changes such as bitemporal concentric visual field defects in cases of an abnormal anatomical relationship between the pituitary gland and the optic chiasma.^18^

## PATHOLOGICAL OCULAR CHANGES

### A. Existing Ocular Pathology Affected by Pregnancy

#### Diabetic Retinopathy

Diabetes is the one of the most common diseases. During pregnancy, diabetic retinopathy (DR) can progress quickly. Aggravation of the condition is dependent on several factors such as the degree of retinopathy at the beginning of pregnancy, how long a patient has been diabetic, glycemic control and comorbid hypertension.^19^

As gestational diabetes carries a very small risk of developing retinopathy, ophthalmologic examination is not necessary. Studies have shown that 10% of patients without DR at the beginning of pregnancy developed nonproliferative changes, and a very small proportion of those patients developed proliferation. Therefore, a baseline examination in the first trimester is sufficient in the absence of visual symptoms.

In patients with nonproliferative DR (NPDR), retinopathy findings during pregnancy showed 50% progression. These findings generally regressed in the third trimester and in the postpartum period. Among patients with severe NPDR, 5-20% transition to proliferative diabetic retinopathy (PDR). Progression of up to 45% can be seen in cases of PDR. However, with pre-pregnancy laser treatment, the risk of progression is reduced by 50%. Pre-pregnancy laser therapy is recommended for patients with PDR or severe NPDR. PDR may regress in the third trimester and postpartum period; these patients require monthly examinations.^20^

Hematologic, hormonal, metabolic, cardiovascular and immunologic factors have pathophysiological roles. Increased retinal capillary blood flow has been shown in diabetic women during pregnancy. It has been posited that this increase may cause endothelial cell damage at the capillary level. During pregnancy, the release of several angiopoietic factors also increases. Progesterone may elevate the production of vascular endothelial growth factor (VEGF) and other angiopoietic factors.^21^

Diabetic macular edema (DME) may develop or worsen during pregnancy. DME is generally observed in pregnant patients with proteinuria or hypertension associated with diabetes.^20^ Laser therapy is recommended for clinically significant macular edema. There are no studies in which treatment was started during pregnancy, and as there have been many cases of spontaneous correction in the postpartum period, observation is sufficient.^22^

#### Tumors

##### Pituitary Adenomas

Previously asymptomatic pituitary adenoma or microadenomas may grow during pregnancy and cause various ophthalmological symptoms. Headache, visual field changes (most frequently bitemporal defect), lowered visual acuity and rarely diplopia may occur. Following pregnancy, adenomas shrink and leave no visual sequelae. In patients with known adenoma, monthly ophthalmologic examination and visual field monitoring is necessary to monitor for tumor growth.^23^

#### Meningioma

Pre-exising meningiomas may vascularize and grow in the second half of pregnancy. It is believed that estrogen and progesterone may be mediators.^24^

#### Uveal Melanoma

Higher ocular melanoma incidence and reactivation rates have been reported in pregnant women compared with nonpregnant women of the same age.^25^ In a later study, a hormonal correlation with uveal melanoma could not be established.^26^

#### Graves’Disease

Graves’ disease is the most common cause of hyperthyroidism during pregnancy. Exacerbation may occur in the first trimester. The condition may subside during the rest of the pregnancy, but become exacerbated again in the postpartum period. Patients require multi-disciplinary monitoring. The preferred medical treatment is propylthiouracil.^3^

#### Toxoplasmosis

Toxoplasmosis is a common infection worldwide, including in Turkey. A primary infection during pregnancy (through transplacental transmission from mother to baby) can result in congenital infection. The fetus is severely effected when the infection occurs in the first trimester; however, transmission to the fetus is more frequently seen in the third trimester, when maternal and fetal circulation are in greatest contact. Latent infections in the mother may become active. With the typical retinochoroiditis presentation, toxoplasmosis is easily diagnosed; however, atypic presentations such as neuroretinitis, papillitis, scleritis and acute retinal necrosis have also been reported.^27^ Oral treatment with the macrolide antibiotic spiramycin is recommended. In the final stage of pregnancy, use of the sulfamethoxazole/trimethoprim combination should be avoided due to the risk of neonatal kernicterus. As current treatment options, use of intravitreal klindamycin (1.0 mg/0.1 ml) and dexamethasone (400 µg/0.1 ml) injections to avoid systemic toxicity has been reported.^28^

#### Multiple Sclerosis

As with other inflammatory conditions, the rate of multiple sclerosis attacks decreases during pregnancy, although it may increase in the first three months postpartum. Optic neuritis attacks may occur as a result of immune-mediated changes during this period. Multiple sclerosis presenting for the first time during pregnancy has also been reported.^29^

#### Pituitary Apoplexy-Sheehan’s Syndrome

Pituitary apoplexy is pituitary gland enlargement due to sudden infarct or hemorrhage in pituitary adenomas. Pregnancy is one of the risk factors for this condition, and occurs as a result of serious postpartum hemorrhage. The condition is a vision-threatening complication and is characterized by sudden headache, vision loss (52%) and visual field loss (64%) and/or ophthalmoplegia. Typical vision field loss is bitemporal superior quadrant defect. Cavernous sinus compression most frequently affects the third cranial nerve, followed by the fourth and less often the sixth. Ptosis, diplopia, anisocoria (midriasis) and lateral-inferior deviation of the globe is seen in cases of third cranial nerve involvement. Horner’s syndrome may also arise as a result of damage to sympathetic fibers.^30,31^

#### Idiopathic Intracranial Hypertension

During pregnancy, idiopathic intracranial hypertension is known to progress with weight gain. The condition occurs with obesity and is characterized by an increase in intracranial pressure of unknown cause. Headache is the most common symptom, and may be accompanied by nausea and vomitting. Ocular findings include blurred vision, scotomas, photopsia, diplopia and retrobulbar pain. Papillary edema is apparent during fundus examination. Keeping weight gain under control is emphasized in treatment.^32,33^

#### Uveitis

During pregnancy, it has been reported that increased endogenic steroids along with multifactorial and complex mechanisms cause both ocular and systemic signs of noninfectious uveitis to subside and attack frequency to decrease.34 Improvements in both the ocular and systemic symptoms of sarcoidosis, spondiloartropathy and rheumatoid arthritis have been observed. However, six months after delivery there may be recurrences.

These pregnancy-related improvements may serve as an advantage for pregnant women with chronic sight-threatening uveitis. It may be possible to reduce or suspend the use of immunosuppressive drugs used to treat uveitis for which data regarding safety is lacking or for which there are known teratogenic effects. Potential attacks can be treated with local corticosteroids.^34^

Vogt-Koyanagi-Harada syndrome, which is characterized by bilateral granulomatous uveitis, exudative retinal detachment, meningeal symptoms, hearing loss and pigment loss has been reported to regress and in some cases complete resolve during pregnancy and the postpartum period.^35^

#### Posterior Scleritis

Posterior scleritis worsens and recurrence increases during pregnancy.^36^ Although the standard treatment is oral steroids, posterior sub-Tenon’s triamcinolone injection is recommended for pregnant patients.^12^

#### Choroidal Neovascularization

There have been case reports of choroidal neovascularization (CNV) during pregnancy. These cases were diagnosed with myopia, punctate inner choroidopathy (PIC), presumed ocular histoplasmosis syndrome (POHS) and idiopathic CNV.^37,38,39^

### B. Ocular Pathology Emerging During Pregnancy

#### Preeclampsia and Eclampsia

In a normotensive pregnant woman, the triad of systemic blood pressure of over 140/90 mmHg, edema and proteinuria after week 20 of pregnancy is defined as preeclampsia. With the addition of contractions without any other cause, the condition is called eclampsia. The incidence of preeclampsia is approximately 5%, and ocular sequelae have been reported in one in three of these patients.36 Though patients frequently complain of blurred vision, they may also experience photopsia, scotoma and diplopia.

Changes in preeclampsia-related retinopathy resemble those in hypertensive retinopathy. The most common finding is retinal arteriolar narrowing, which is usually focal but may also be generalized. Other changes include retinal hemorrhage, edema, exudate, nerve fiber layer infarcts and intravitreal hemorrhage secondary to neovascularization. There is a positive correlation between the severity of preeclampsia and the degree of retinopathy. Most of these findings return to normal following the resolution of preeclampsia. Cases of preeclampsia-related retinopathy with underlying diabetes, chronic hypertension and kidney disease may be more severe.

Optic nerve findings in preeclampsia are papillary edema, ischemic optic neuropathy and optic atrophy. Exudative retinal detachment is seen in 1% of preeclamptic patients and 10% of eclamptic patients.^36^ In a Turkish study by one of the authors of this manuscript (RBK) including 47 patients with preeclampsia, 3 patients exhibited exudative retinal detachment; two of the cases resolved spontaneously following birth, while one required postpartum systemic steroid treatment due to very low visual acuity.^40^

#### Central Serous Chorioretinopathy

Pregnancy is believed to be a risk factor for the development of central serous chorioretinopathy (CSCR). It is most frequently seen in the third trimester, though it may also appear in the first and second trimesters. In one study, 90% of pregnant CSCR patients had fibrinous subretinal exudate, while this rate was 20% in nonpregnant CSCR patients.41 Diagnosis is easily made with posterior segment optical coherence tomography. Spontaneous regression is observed at the end of pregnancy or after birth; however, there may be a tendency for recurrence in the same eye in subsequent pregnancies.^41,42^

#### Vascular Occlusive Diseases

This group of conditions includes retinal artery occlusions, retinal vein occlusions, disseminated intravascular coagulopathy (DIC), thrombotic thrombocytopenic purpura (TTP), antiphospholipid antibody syndrome (APS), amniotic fluid embolism, and cerebral venous thrombosis. As indicated previously, hypercoagulability occurs in pregnancy. Pregnancy-related central and branch retinal artery occlusion has rarely been reported. Retinal vein occlusions are rarer than arterial occlusions.

DIC may develop in pregnancies with complications such as abruptio placenta, preeclampsia/eclampsia, complicated birth, amniotic fluid embolism, intrauterine infection and intrauterine death. DIC is a serious condition characterized by diffuse small vessel thrombosis and subsequent hemorrhage and tissue necrosis.^43,44^ In the eye, the choroid layer is most affected; thrombosis in the choriocapillaris disrupting the retinal pigment epithelium may cause serous retinal detachment. Ocular symptoms improve with DIC treatment, though mild pigmentary changes may persist.^45^

HELLP syndrome is a condition characterized by hemolysis, elevated liver enzymes and lowered thrombocyte count; it is typically seen in preeclamptic patients and generally appears with DIC. Serous retinal detachment, vitreous hemorrhage, central retinal vein occlusion and Purtscher-like retinopathy have been reported in these patients.^46,47,48^

TTP is a rare disease, and ocular changes are observed in 10% of TTP patients. Fundus changes occur in the form of serous retinal detachment, retinal hemorrhage, exudates and narrowing of the arterioles. Involvement of the vessels supplying the optic nerve may lead to optic atrophy. Anisocoria, subconjunctival hemorrhage, scintillating scotoma, extraocular muscle paresis and homonymous hemianopia may occur.^49^

APS is a thrombophilic condition; patients are predisposed to arterial and venous thrombosis and antiphospholipid antibody-related pregnancy morbidity is observed. Anterior segment findings accompanying this syndrome may include conjunctival telangiectasia and microaneurysms, episcleritis, limbal or filamentous keratitis, and iritis. Posterior segment findings may include vitreitis, retinal detachment, posterior scleritis, central retinal vein occlusion, retinal vein branch occlusion, cilioretinal artery occlusion, increased venous tortuosity, retinal hemorrhage, and soft exudates. Furthermore, vascular thrombosis may develop in the choroid, optic nerve, visual pathways and ocular motor nerves.^50,51^

Amniotic fluid embolism, though rare, is a serious condition with fatal complications; 85% of cases end in mortality. Symptoms include chills, cyanosis, convulsions and shock. The optic nerve, visual pathways and occipital cortex may be affected and central retinal artery occlusion may develop.^52^

Risk of venous and sinus thrombosis increases during pregnancy due to hormonal changes. Between 5-20% of cerebral venous thrombosis cases are women who are pregnant or in the postpartum period and papilledema is found in 35% of these cases.^53^

#### Medication Use During Pregnancy

Regardless of attempts to avoid or postpone the use of medication during pregnancy, in some cases it cannot be avoided. It is therefore essential that physicians are well informed of the potential teratogenic effects of the medications being prescribed. Based on available clinical studies and experience, the U.S. Food and Drug Administration (FDA) has classified drugs into five categories (A, B, C, D and X) according to the severity of potential teratogenic effects.^54^

#### Food and Drug Administration Categories:

##### - Category A:

Adequate and well-controlled studies did not show a risk to the fetus in the first trimester of pregnancy, and there are no data suggesting a risk in the second and third trimesters. Category A is the safest category.

##### - Category B:

Animal studies did not show a risk to the fetus, but there are not adequate and well-controlled studies in pregnant women. Alternatively, adverse effects appeared in animal studies, but adequate and well-controlled studies with pregnant women did not reveal a risk to the fetus during any trimester. Category B drugs can be used if necessary.

##### - Category C:

Adverse effects were observed in animal studies, and adequate and well-controlled studies in humans have not been conducted. However, the potential benefits may justify the use of the drug in pregnant women despite the potential risks.

##### - Category D:

There is evidence indicating risk to the human fetus based on adverse reaction data from investigational or marketing experience, but the potential benefits may justify the use of the drug during pregnancy despite the potential risks. The drug may be used with caution if the mother and fetus face greater risks from not using it.

##### - Category X:

Fetal abnormalities were seen in animal or human studies and/or there is evidence indicating risk to the human fetus based on adverse reaction data from investigational or marketing experience. The drug’s risks outweigh the potential benefits of its use during pregnancy. Use is not recommended.

#### Use of Ophthalmic Drugs During Pregnancy

As a general principle, to limit systemic absorbtion and avoid toxicity, the lowest dose possible should be given and after administering eye drops the patient should perform punctal occlusion by applying pressure to the nasolacrimal canal and wipe away excess to prevent systemic absorption. There is a lack of in-depth research regarding the effects of ophthalmologic drugs on pregnancy and lactation.^55^

#### Drugs Used in Diagnostic Tests

Fluorescein crosses the placenta and is a category C drug. Indocyanine green does not cross the placenta, but is also in category C.

Tropicamide, cyclopentolate and epinephrine, used for dilatation and cycloplegic purposes, are category C drugs. Although there are no reports of teratogenic effects from topical use, minor fetal malformations have been reported from systemic use of phenylephrine, atropine and homatropine, and there is relative contraindication for their ophthalmologic use.^56^ In the first three months of pregnancy, even their application for the purpose of examination is not recommended.

#### Antibiotics

Chloramphenicol, neomycin and tetracycline must not used during pregnancy. Erythromycin, ophthalmic tobramycin, ophthalmic gentamicin, polymyxin B, acyclovir and quinolones are reported to be safe.^56,57^ Systemic tobramycin is a category D drug, topical tobramycin is category B, netilmicin and tetracycline are category D, and all other antibiotics used locally are category C. Among antiviral drugs, trifluridine is category C and acyclovir is category B.

#### Anti-inflammatory and Anti-allergy Drugs

In general, the potential risks and benefits must be considered when prescribing these drugs. Systemic corticosteroids are contraindicated due to their teratogenic effects and their role in CSCR; topical steroids have no known teratogenic effects. All topical steroid and nonsteroid anti-inflammatory drugs are in category C. Topical cyclosporine is in category C. Among topical antihistamines and anti-allergy drugs, ketotifen, olopatadine and epinastine are category C drugs; sodium cromoglycate and emedastine are in category B.^54^

#### Glaucoma Medications

Beta-blockers, prostaglandin analogues, carbonic anhydrase inhibitors and miotics are category C drugs. Beta-blockers should be used with caution in the first trimester and be discontinued a few days before birth to prevent neonatal beta blockade. As beta-blockers are concentrated in breast milk, they should not be used while lactating.^57^

Systemic and topical carbonic anhydrase inhibitors have teratogenic and hepatorenal effects and are therefore contraindicated both in pregnancy and during lactation.^58^ The use of miotic drugs during pregnancy appears to be safe. Prostaglandins may cause early delivery or miscarriage. In a study including 11 pregnant women using latanoprost, 1 patient miscarried, 1 patient did not continue follow-up, and 9 patients completed their pregnancies without complications.^59^ Brimonidine, an alpha-2 agonist, is in category B. However, maternal use during the lactation period carries the risk of apnea and bradycardia in the newborn^60^

#### Anti-Vascular Endothelial Growth Factor Agents

Anti-VEGF agents are used to treat PIC, POHS, miopic CNV, diabetic and uveitic macular edema, and retina and iris neovascularization in PDR in patients of child-bearing age. Although systemic absorption is very low and bevacizumab cannot cross the placenta due to its high molecular weight (149 kD), it should be kept in mind that it can theoretically affect placental vasculature. Among anti-VEGF agents, bevacizumab and ranibizumab are category C and pegaptanib is category B.

There are case reports in the literature regarding the use of anti-VEGF intravitreal injection during pregnancy.37,39 One case underwent photodynamic therapy (PDT) in the first two weeks of pregnancy and received 1.25 mg intravitreal bevacizumab in the third month; the pregnancy was completed with no problems.^37^ Another case received intravitreal bevacizumab in the third trimester and encountered no problems. A patient with idiopathic choroidal neovascularization received ranibizumab in the third trimester and also experienced no problems.39 In one study, four pregnant patients received an average of 2.5 intravitreal injections (range, 1-6) of bevacizumab with no adverse effects, while in another report, two patients with unknown pregnancies miscarried following bevacizumab treatment in the first trimester.^38^

#### Verteporfin

Verteporfin is a category C drug. Anophthalmia and microphthalmia have been reported in rats exposed during organogenesis to verteporfin at 40 times the dose used in humans.^37^ However, two patients with unknown pregnancies underwent PDT at 3 and 12 weeks post-conception and later gave birth to healthy babies.^61,62^

#### Anesthetic Agents

For pregnant patients for whom ocular surgery is unavoidable, the safest anesthetics may be lidocaine and citanest, which are category B drugs.54 They may be used as sub-Tenon’s and peribulbar anesthesia. Bupivacaine, a category C drug, is not recommended due to the risk of bradycardia in the fetus. Proparacaine hydrochloride, which is used as a topical anesthetic, is also in category C.^54^

## CONCLUSION

Visual complaints are a common occurrence in pregnant women. It is extremely important that ophthalmologists are aware of the various physiological and pathological conditions that may arise or be altered during pregnancy, and are knowledgeable about their differential diagnosis, treatment and monitoring. Especially rare and serious complications may include visual symptoms and pregnant patients may initially be seen by an ophthalmologist. Responding carefully, rapidly and strategically to patients with these complaints can prevent possible risks to mother and baby.
